# Patient-identified priorities for successful partnerships in patient-oriented research

**DOI:** 10.1186/s40900-022-00384-4

**Published:** 2022-09-07

**Authors:** Maria J. Santana, D’Arcy Duquette, Paul Fairie, Ingrid Nielssen, Sumedh Bele, Sadia Ahmed, Tiffany Barbosa, Sandra Zelinsky

**Affiliations:** 1 Department of Pediatrics, Cumming School of Medicine, University of Calgary, Calgary, AB Canada; 2 Department of Community Health Sciences, Cumming School of Medicine, University of Calgary, Calgary, AB Canada; 3 Alberta Strategy for Patient-Oriented Research Patient Engagement Team, Edmonton, Canada; 4 Patient Partner, Alberta SPOR SUPPORT Unit, Edmonton, Canada

**Keywords:** Patient and public involvement, Co-production, Patient priorities

## Abstract

Albertans4HealthResearch, supported by the Alberta Strategy for Patient-Oriented Research Patient Engagement Team, hosted a virtual round table discussion to develop a list of considerations for successful partnerships in patient-oriented research. The group, which consists of active patient partners across the Canadian province of Alberta and some research staff engaged in patient-oriented research, considered advice for academic researchers on how to best partner with patients and community members on health research projects. The group identified four main themes, aligned with the national strategy for patient-oriented research (SPOR) patient engagement framework, highlighting important considerations for researchers from the patient perspective, providing practical ways to implement SPOR’s key principles: inclusiveness, support, mutual respect, and co-building. This commentary considers the process behind this engagement exercise and offers advice directly from active patient research partners on how to fulfill the operational patient engagement mandate. Academic research teams can use this guidance when considering how to work together with patient partners and community members.

## Plain English summary

Albertans4HealthResearch, supported by the Alberta Strategy for Patient-Oriented Research (AbSPORU) Patient Engagement Team, hosted a virtual round table discussion to develop a list of considerations for successful partnerships in patient-oriented research. The group, which consists of active patient partners across the Canadian province of Alberta and some research staff engaged in patient-oriented research, considered advice for academic researchers on how to best partner with patients and community members on health research projects. The group identified four main themes, aligned with the national Strategy for Patient-Oriented Research (SPOR) Patient Engagement Framework, highlighting important considerations for researchers from the patient perspective, providing practical ways to implement SPOR’s key principles: inclusiveness, support, mutual respect, and co-building. This commentary considers the process behind this engagement exercise and offers advice directly from active patient research partners on how to fulfill the operational patient engagement mandate. Academic research teams can use this guidance when considering how to work together with patient partners and community members.

## Background

The Albertans4HealthResearch (AB4HR) Collaborative Council identified a series of considerations for successful partnerships in patient-oriented research during a virtual round table held in Fall 2021. The Council is a team with diverse expertise and lived experiences, including active patient partners and community members (that is, people with lived experience of health conditions and/or health care who have experience working in health research), from across the Canadian province of Alberta. The round table discussion was guided by the question “What are the “Do’s and Don’ts” when partnering in health research?”.

The role of patients as partners in health research in Canada has been highlighted since the Canada’s Strategy for Patient-Oriented Research (SPOR) was established by the Canadian Institutes of Health Research (CIHR) in 2015. SPOR emphasizes the importance of patients, researchers, health care providers and other decision-makers working together as active and equal partners on the research that aims to improve health care and health outcomes for all Canadians [1]. While there are guidelines to patient-oriented research written from an academic perspective for researchers [2–5], there are fewer resources to guide research teams written from the patient and community perspective. Therefore, this manuscript depicts the advice that a diverse group of patients and community members want to share with academic researchers when they partner with them in health research. This paper is written with two patient partners who are leaders of this Council, and the intention is to faithfully represent what the Council wanted academic researchers to consider when working together.

## The collaborative council

The AB4HR Collaborative Council is a provincial team comprised of individuals with diverse expertise, lived experiences, coming from diverse socio/cultural backgrounds, living in Alberta, Canada. Membership includes those living with unique chronic health conditions, academic and clinical researchers, members of patient and community organizations, and stakeholders from non-profit, government and other health service organizations. The Council meets quarterly to discuss issues related to POR, to bring research priorities to the discussion table, and to explore novel approaches and solutions focused on improved healthcare policy and practice for all Albertans. The Council is supported by the Alberta SPOR SUPPORT Unit’s Patient Engagement Team, which consists of expert staff who support patient partners and researchers across the province. Specific opportunities to work together to identify research priorities, to collaborate on grant applications and to work in meaningful partnerships in additional roles in POR projects are described on the public facing website.

## The process

The round table discussion of 24 members held in September 2021 was guided by the question, “What are the ‘Do’s and Don’ts’ when partnering in health research?” Small group discussions happened in breakout rooms consisting of random selection of 4–5 people. A Google Jamboard was used for each contributing member to post as many ‘Do’s and Don’ts’ as they felt were important to positive experiences on POR teams. After 20 min of posting, the larger group of 24 people reconvened. The sticky notes were all shared with the larger group for review and discussion. There was a final step that included merging some of the themes (refining and avoiding repetition) and reviewing the content to ensure that all insights were appropriately captured and reflected.

Subsequently, members of the Patient Engagement Team copied these notes to a master Excel table and completed a deductive thematic analysis, aligning the content with the four CIHR SPOR Guiding Principles of patient engagement as identified in the CIHR SPOR Patient Engagement Framework [1]. These principles are *inclusiveness, support, mutual respect, and co-build* [1]. (Table 1 describes the principles in more detail.)

**Table 1:** The CIHR SPOR patient engagement principles [1]

*Inclusiveness* integrates a diversity of patient perspectives and reflects their contributions and lived experience	*Support* is provided to patient partners to enable them to fully contribute to discussions and decisions related to the health research project. This includes creating safe environments that promote honest interactions, addresses cultural competence, provides training and education, and offers financial compensation for their involvement	*Mutual respect* happens when all members of the research team (academics, clinicians, and patients) acknowledge and value each other's expertise and experiential knowledge	*Co-build* sees patients, researchers, and clinicians working together throughout a project to identify gaps, set priorities for research, and partner to produce and implement solutions to improve health and health care

The analysis was then shared back with the Collaborative Council, and we report the results in the following section.

## The results

Based on the descriptions in Table 1, the themes were classified into these four guiding themes, aligned with the Guiding Principles found in CIHR’s Patient Engagement Framework described In Table 1.

We have summarized each theme and then synthesized the feedback from the Council into patient-driven considerations for researchers engaged in patient-oriented research projects in subthemes.

### Theme 1: inclusiveness

The Council highlighted three themes: diversity, cultural sensitivity, and tokenism.

#### Subtheme 1a: diverse experiences and perspectives

Patient partners come from a variety of backgrounds. Diversity can exist across populations and communities, and within and across patient experience and disease. Researchers should consider how to embrace this essential, wide-ranging diversity, such as accommodating different comfort levels with project involvement, language, as well as differences in values and worldviews. Each person and the abilities and issues they experience are unique. Identifying and offering supports for patient partners to collaborate comfortably and equitably on health research teams should be done at an individual level. In addition to accessibility barriers, patient partners living in rural areas may require extra considerations such as time and costs to travel or to access high-speed internet.

Include the perspectives of others in decision-making. All insights and points of view are relevant to the project. Get to know your partners, their motivations, backgrounds, and skillsets, and communicate in ways appropriate to them. Recruit thoughtfully. It is important to consider both established and innovative, approaches to inviting, including, and making welcome multiple experiences and perspectives.

#### Subtheme 1b: cultural sensitivity

Cultural sensitivity supports the inclusion of patient partners that are not usually part of health research teams. To meaningfully partner with individuals and their communities, we need to create and sustain inclusive and safe spaces to work together.

#### Subtheme 1c: tokenism

Avoid tokenism by thoughtful inclusion of the patient voice in project design. Actively engage with patient partners throughout the whole research cycle (from generating the research question to disseminating the findings) and not when the project is already in implementation as that might be too late. Also include patient partners in dissemination and feedback loops when the project is complete.

### Theme 2: support

The Council highlighted four themes: tailoring support, planning and preparation for engagement activities, compensation and reimbursement, and sustainability.

#### Subtheme 2a: tailoring support

*Tailoring support* means identifying then addressing barriers, including language, digital access, and other issues that may hurt equitable partnerships. Translation, interpretation, and other services can help with language barriers and literacy challenges, these are not always available in a timely fashion. Patient partners should be provided with mentorship and capacity building opportunities to practice and hone the skills (e.g. research skills training) they are expected to use during the project. Tailoring supports facilitates the planning and preparation towards working together.

#### Subtheme 2b: planning and preparation

Planning and preparation require understanding and addressing the diverse motivations and the expectations that patient partners have when engaging in health research projects. Roles and timelines should be discussed and clearly laid out as soon the partnership is acknowledged. These may include discussions about capacity and commitment to join the research team. During the project planning and design researchers should demonstrate cultural sensitivity and learn intercultural communication. Additionally, researchers should be flexible in setting times for meeting with patient partners. These times might be outside standard work hours, such as evenings or weekends. Consider holding meetings in neutral, inclusive spaces. Technological support (such as hardware and software) should be offered together with the support needed for their use.

#### Subtheme 2c: compensation and reimbursement

Offering compensation and reimbursement is a vital to supporting individuals to partner in health research. While planning to partner with patients, researchers need to recognize and offer appreciation for time and contribution (compensation) of patient partners and reimbursement. Researchers must consider these budgetary considerations and include them in the grant applications. The amount and mode (e.g., gift cards, e-transfers, etc.) of compensation or reimbursement must be discussed with patient partners at the start of the collaboration.

#### Subtheme 2d: sustainability

Sustained support includes addressing patients’ motivations, expectations, and capacity and checking in on a regular basis. Patient engagement takes time and additional funds, so researchers should develop a plan that ensures patient engagement can be sustained, especially for multi-year projects. The same group of patient partners can be part of multiple small studies within a larger research project, which can help researchers avoid reinventing the wheel. Researchers should also budget for ongoing engagement and regular evaluation. Support provided to patient partners should be regularly reviewed.

### Theme 3: mutual respect

The Council highlighted four themes: roles and responsibilities, respectful collaboration, communication and planning, and sharing knowledge.

#### Subtheme 3a: roles and responsibilities

Roles and responsibilities should be discussed early on with all members of the research team, including patient partners. These discussions can help to identify individual strengths, interests, and availability of time to commit to the project. Roles and responsibilities may evolve depending on the needs of the project and the engagement of the team.

#### Subtheme 3b: respectful collaboration

Respectful collaboration, or mutual respect, defined by the Canadian Institutes for Health Research as “[r]esearchers, practitioners and patients acknowledge and value each other's expertise and experiential knowledge” [1], is key to mutual respect to gain an understanding from all members of the research team with a focus on confidentiality and ethical approaches to working together.

#### Subtheme 3c: communication and planning

Take the time to establish processes that will help the team to work together more efficiently including taking the time to introduce all team members to each other and identify their roles, creating a contact list with name, role, location, and contact information. Consider patient partners taking co-chair roles at team meetings, patient partners could help to co-plan and distribute meeting agendas and take on facilitation and host roles at meetings. It is important to consider asking for input from all team members and try to provide feedback on team members’ contributions and involvement throughout the partnership.

#### Subtheme 3d: sharing knowledge

Knowledge sharing can help us to learn from one another, understand worldviews different from our own, and build mutually beneficial relationships. Learning from each other helps to ensure that all team members have been provided information to contribute more meaningfully to the project work. This can include sharing details about the research (e.g., proposal, timelines), creating a document that describes acronyms that are commonly used, and understanding how the skill sets and expertise of all team members can advance the project.

### Theme 4: co-build

The Council highlighted three themes: building and maintaining relationships, tokenism, and evaluation.

#### Subtheme 4a: building and maintaining relationships

Recognize that patient partners are equal members of the research team and that they bring valuable knowledge and expertise based on their lived, professional, or other experience. Respectful collaborations and relationship building take time—consider including time for an icebreaker at the beginning of each meeting to get to know one another and make sure to include extra time to co-build and work together. Working together to establish project goals and objectives will help to ensure that all perspectives are heard so that priorities important to patients, families, and communities help to inform the aim of the study early on. Consider going over the project goals and objectives at each stage of the research cycle.

#### Subtheme 4b: tokenism

Engage patients as equal partners early in the grant application process and throughout the research cycle will help to provide opportunities to learn from each other, to co-develop project aims and goals, and to work better together throughout the study.

#### Subtheme 4c: evaluation

Patient partners should be involved in the process of evaluating the project. Evaluation was seen by the patient partners as necessary to understand the outcomes of the research project and any implications that these outcomes may have at clinical level.

## Conclusions

Engaging patients in the health research that informs health care practice and policy has positive outcomes for patients, researchers, research projects and health care [2, 5]. Authentic relationships are those built on trust and over time, and are essential elements to effective research team collaborations [3, 5, 6]. These considerations for successful partnership between patient partners and academic researchers in health research represent the perspectives of individuals committed to POR in the Canadian health research landscape.

The AB4HR Collaborative Council members leveraged long-standing relationships and established collaborative spaces to actively reflect on their POR experiences—what has worked well, and what could be improved, to assure meaningful and authentic patient partner collaboration on POR projects. The resulting themes were aligned with the four principles of patient engagement identified in the CIHR SPOR Patient Engagement Framework that defines patients as active and equal partners on health research teams, engaged early, often and at as many stages of the CIHR Research Cycle Figure [7]. (The ‘4’ in the Albertans4HealthResearch name reflects the four of these principles.) While the advice from the Council may been developed in a specific Alberta context, they align and add to previous consultations and work about patient and public engagement in health research [8–10].

In Canada, we have made tremendous advances in working with patients, bringing their lived experiences to make health research more relevant to them, but we still have a long way to go. To work together in health research, we need to invite as many multiple voices and perspectives as possible, and from those with different, and often dynamic, backgrounds and contexts to sit at shared research tables and engage in reciprocal, respectful and generative ways. We need more time to all learn how to best work together and considering this advice from active patient researchers into account when planning and preparing for health research will help to make that possible, understanding the support needed to build the trust and create an environment in which we can partner meaningfully in mutual respect allowing for co-building health research projects that are relevant to all.

## Data Availability

Not applicable.

## Abbreviations

AB4HR: Albertans4HealthResearch
CIHR: Canadian Institutes for Health Research
SPOR: Strategy for patient-oriented research
POR: Patient-oriented research
